# Supplementary material for: Bacterial community dynamics are linked to patterns of coral heat tolerance
Source: Nat Commun. 2017 Feb 10;8:14213. doi: 10.1038/ncomms14213 (PMC5309854; doi:10.1038/ncomms14213)
Supplement: Supplementary Information — Supplementary Figure [file ncomms14213-s1.pdf]

Supplementary Figure

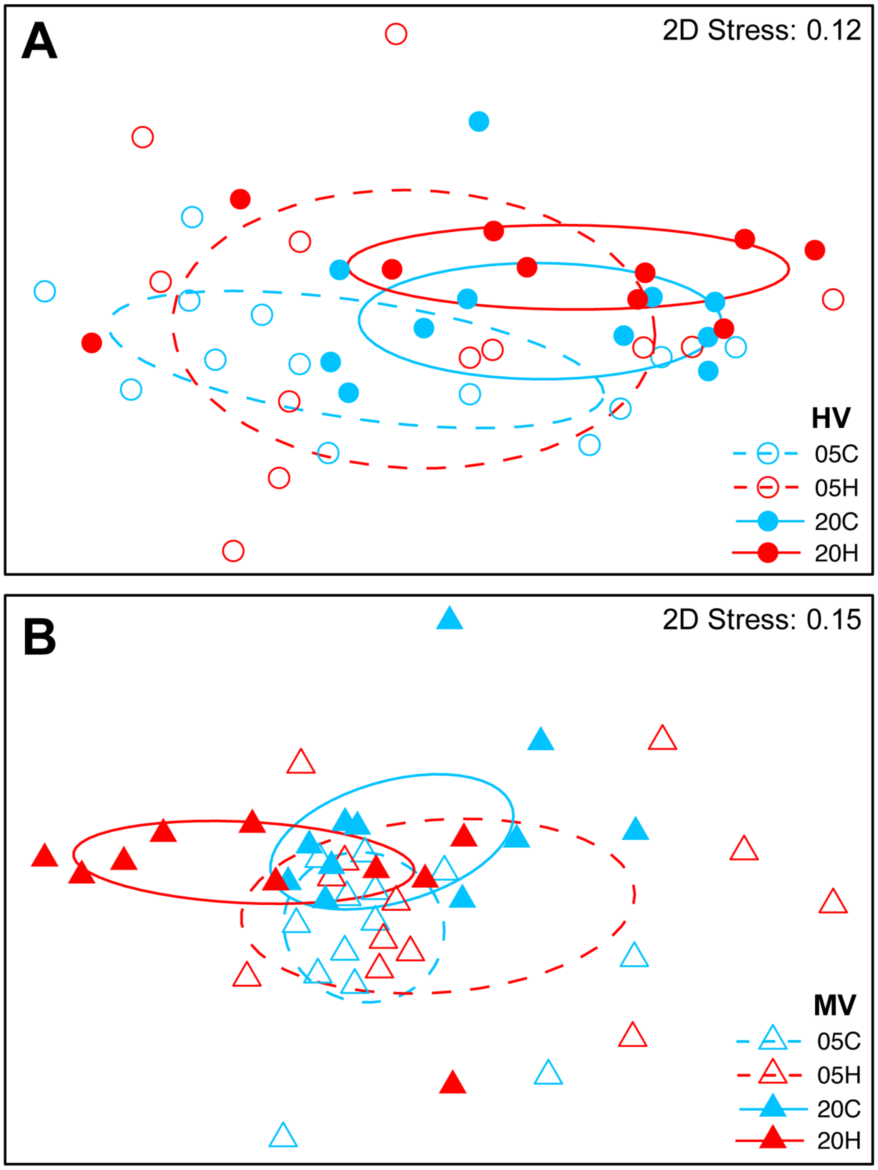

Supplementary Figure 1 Bacterial community composition of *Acropora hyacinthus* after a thermal bleaching experiment based on non-metric multidimensional scaling (nMDS) ordination. (A) Bleaching resistant corals from the highly variable (HV) pool maintain stable microbial communities during short-term heat stress (ANOSIM, $P > 0.05$); (B) Microbial communities in bleaching-susceptible corals from the moderately-variable (MV) pool change significantly at 20 h during a short-term heat stress experiment (ANOSIM, $P < 0.05$). 05C and 20C = 5 h and 20 h control short-term heat stress experiment; 05H and 20H = 5 h and 20 h treatment short-term heat stress experiment.
